# Effects of a case-based interactive e-learning course on knowledge and attitudes about patient safety: a quasi-experimental study with third-year medical students

**DOI:** 10.1186/s12909-016-0691-4

**Published:** 2016-07-11

**Authors:** Rainer Gaupp, Mirjam Körner, Götz Fabry

**Affiliations:** Medical Faculty, Medical Psychology and Medical Sociology, Freiburg University, D-79104 Freiburg, Germany

**Keywords:** Patient safety, Medical education, Attitudes, Safety culture

## Abstract

**Background:**

Patient safety (PS) is influenced by a set of factors on various levels of the healthcare system. Therefore, a systems-level approach and systems thinking is required to understand and improve PS. The use of e-learning may help to develop a systems thinking approach in medical students, as case studies featuring audiovisual media can be used to visualize systemic relationships in organizations. The goal of this quasi-experimental study was to determine if an e-learning can be utilized to improve systems thinking, knowledge, and attitudes towards PS.

**Methods:**

A quasi-experimental, longitudinal within- subjects design was employed. Participants were 321 third-year medical students who received online surveys before and after they participated in an e-learning course on PS. Primary outcome measures where levels of systems thinking and attitudes towards PS. Secondary outcome measures were the improvement of PS specific knowledge through the e-learning course.

**Results:**

Levels of systems thinking showed significant improvement (58.72 vs. 61.27; *p* < .001) after the e-learning. Student’s attitudes towards patient safety improved in several dimensions: After the course, students rated the influence of fatigue on safety higher (6.23 vs. 6.42, *p* < .01), considered patient empowerment more important (5.16 vs. 5.93, *p* < .001) and realized more often that human error is inevitable (5.75 vs. 5.97, *p* < .05). Knowledge on PS improved from 36.27 % correct answers before to 76.45 % after the e-learning (*p* < .001).

**Conclusions:**

Our results suggest that e-learning can be used to teach PS. Attitudes towards PS improved on several dimensions. Furthermore, we were able to demonstrate that a specifically designed e-learning program can foster the development of conceptual frameworks such as systems thinking, which facilitates the understanding of complex socio-technical systems within healthcare organisations.

## Background

Medical errors are a significant cause of morbidity and mortality, even in highly developed healthcare systems [1–4]. Medical errors lead to approximately 100,000 deaths in the USA [2] and to 18,000 deaths in Germany [5] annually. Therefore, improving patient safety is a major goal in healthcare systems worldwide [6]. In recent years, several initiatives on national [7] and international [6] levels have aimed to design patient safety curricula and to define learning objectives for both under- and postgraduate students [8–10]. Because of the complexity of the construct of patient safety, such curricula often cover a broad spectrum of content, ranging from simple skills to rather complex competencies [11]. Improving patient safety requires systems-level interventions within a healthcare organization [2, 12–15], and it is widely accepted that knowledge alone will not lead to sustainable improvements in patient safety; instead, a holistic approach that includes behavioural and affective interventions is necessary for such improvements [16]. In this regard, educational approaches to patient safety often include conceptual frameworks such as the systems approach [17]. Systems thinking can be considered a meta strategy to improve patient safety, because errors in medicine often have multifactorial causes and must be approached from various levels of the system to be understood [2, 16, 18, 19]. Affective interventions, an essential aspect of patient safety, require a methodical approach that leads to deep reflections on the learner’s norms and values [20]. Action- or discussion—based learning methodologies such as patient simulation accompanied by structured debriefings are frequently deployed to achieve the triad of knowledge, conceptual frameworks and affective objectives [14]. Although such methodologies have proven effective [21], they remain resource-intensive in terms of money, time and faculty [22]. Digital learning solutions might overcome or at least decrease this problem by reducing required “face time” [23]. Web 2.0 technology allows the development of interactive learning environments that can provide a systematic approach to knowledge acquisition. Following the principles of problem-based-learning (PBL) [24], case studies, video sequences or specifically designed dilemmas, e-learning modules may trigger reflections of the learner’s values, beliefs and attitudes. In so doing, such modules prepare the learner for future learning processes in face-to-face action-oriented teaching sessions or clinical settings [25].

### ELPAS — eLearning Patient Safety

Although e-learning has become a standard teaching approach in medical education internationally [26] and is employed for a wide variety of subjects and disciplines [25, 27–29], it is still rarely used to approach patient safety subjects [17]. In Germany, teacher-centred approaches (i.e. lectures) are still widely used although more and more medical schools introduce learner-centred formats such as problem- or team-based learning. The German Council of Science and Humanities promotes this development of student centred learning to foster critical thinking, systems thinking, decision making or communication skills [30]. Against this background we developed an online course on patient safety aligned with the principles of problem-based-learning [31], and implemented it in the third year of the undergraduate medical curriculum. The mandatory online course was integrated into a more comprehensive curricular module on “health economics, the healthcare system and public health”. Content for the e-learning course was defined based on the WHO patient safety curriculum [6]. Because the course is the first instance where students at our institution are formally taught about issues of patient safety, we decided to focus on general aspects of patient safety, i.e. teamwork [32], situational awareness [33, 34], and error management [16, 18, 19].

We chose a work process-oriented approach [35] for both content selection and sequencing to ensure practical relevance of modules. However, we did not only focus on processual knowledge, but rooted the modules in conceptual models, e.g. the big five model of teamwork [32], the London Protocol [19] and Endsley’s situational awareness model [33].

Guided by the principles of problem-based-learning [36], we developed interactive online learning modules for each of the three subjects (teamwork, error management, situational awareness). Each module starts with an ill structured clinical problem to trigger interest and stimulate elaboration [31]. In the light of the contextual dependency of learning [36], we chose emergency medicine and anaesthesiology scenarios, as the students enrolled in our e-learning course did simultaneously attend a hands-on course on emergency medicine. Activating prior knowledge acquired in this course facilitates the problem solving process for cases presented in the patient safety online modules. By integrating several educational technologies, each module was designed to foster self-regulated learning, provide resources for problem solving and allow small group collaboration. To foster self-regulated learning, the modules support monitoring strategies [37] by providing self-assessment tools, quizzes and checklists. Through learning material, videos, podcasts and literature links, each module provides a profound source for problem solving. Such knowledge repositories are not only an important element of PBL, but support self-regulated learning, as they support students’ resource management strategies [38]. Web 2.0 technology integrated in the modules allows synchronous and asynchronous online collaboration through discussion boards, chat rooms and etherpads. Through these educational technologies, students elaborate their learning outcomes, reflect the clinical problems and develop a problem statement in a collaborative learning process. Table 1 provides an overview of the cases used for each module.

In all modules, clinical scenarios and multi-perspective tasks were used to foster systems thinking; however, a specific focus on the effects of systems thinking was placed in the error management module [19]. Video case studies [39] were used to demonstrate and practice systems-level error analysis [40] but also to challenge existing values and norms, as those videos showed lethal, but realistic consequences of errors in various parts of the system. The e-learning course was designed to allow asynchronous and location-independent learning via a browser-based online platform (ILIAS Vers. 5.1, General Public Licence). Although the online platform supports responsive designs and the e-learning can be accessed on mobile devices, the course is optimized for use on laptop or desktop computers. As the ILIAS platform is being used throughout the medical curriculum, students are aware of the functionality of the system. Participants were invited to the online platform via e-mail and could access the course by using their student ID and password.

ELPAS is the result of a local initiative to increase students’ awareness to patient safety. The development of the online modules was funded by internal funds of the department. The development of the course including the development of specific learning material (i.e. videos, podcasts, interactive texts etc.) was done without external service providers to keep costs low, all developments were done by one research fellow (RG), for regular reviews of the course we used a team of 4 persons. In total, four months developing time was spent on the course.

### Goal and research questions

The major goal of this study was to identify the impact of the described e-learning module dealing with several aspects of patient safety on students’ domain-specific knowledge, awareness and attitudes and systems thinking. Because a relevant subgroup of medical students in Germany already has professional experience in various fields of the healthcare system (e.g. nursing, emergency medical services), a sub-analysis aimed to identify effects of such professional experience on attitudes and knowledge. Finally, because both the learning content of the intervention and the assessment objectives focus on systems-level interdependences in healthcare organizations, we expect correlations between the level of systems thinking and specific objective (knowledge post-test) as well as subjective (ratings of the e-learning course) outcome measures. To understand, whether students perceive the programme as valuable and user-friendly the study includes a satisfaction and usability questionnaire.

## Methods

A quasi-experimental, longitudinal within-subjects design was employed. Participants were 321 third-year medical students who received online surveys before and after they participated in the mandatory e-learning course on patient safety. The online course was conducted between October 2015 and December 2015. There were no parallel courses in this period that focused on patient safety, nor did students attend clinical clerkships within the study period where issues of patient safety might have been discussed. Students were recruited via email, and we used the survey functionality of a learning management system (ILIAS, Vers. 5.1, general public licence) and student IDs to create a paired dataset, containing pre- and post-test data, as well as outcome results.

In the pre-test we measured attitudes toward patient safety, using the German version of the Attitudes to Patient Safety Questionnaire (GAPSQ) [41] and the extent of systems thinking, using the Systems Thinking Scale (STS) [42]. Both surveys have been used in previous research [41, 43], and both have been validated [41, 42] in the field of medical or health science education. The GAPSQ [41] contains 7 dimensions (see Table 2 for dimensions and items), measured on 7-point Likert scales (1 = lowest value). Its internal consistency reliability has been tested by its creators (ɑ = 0.74) [41]. In our study, the reliability of all subscales was also good (ɑ = .73 to ɑ = .93 in pre- and post-test data), except for the subscale *error inevitability* (ɑ = .37). For this scale, the two single items were retained for further analysis; the means of all other subscales were summed to produce a total score. The STS contains 20 frequency-rating items (e.g. “I think of the problem at hand as a series of connected issues”; “I keep in mind that proposed changes can affect the whole system”). Response options range from 0 (I never do) to 4 (I almost always do); the STS score is calculated by summing all items (possible range: 0 – 80 points). Good test-retest reliability has been reported by the authors (*n* = 36, *r* = .74) [26]. In our study, the scale showed high internal consistency (ɑ = .82 to .88 in pre- and post-test data). Knowledge of patient safety was measured by four multiple-choice questions, each presenting a short case. The resulting knowledge score had a range from 0 % (no correct answers) to 100 % (4 correct answers).

**Table 1:** Case based approach to ELPAS online modules

Module	Case	Technology used	Learning outcome
Team work	Case presented by video: A CPR-team resuscitates a patient, teamwork is not optimal.	Online video.	Students apply Big-5 model of teamwork to find solutions.
		Etherpad to work on the problem analysis collaboratively.	
Error management	A real case from a critical incident reporting system (CIRS) is reported.	Data repository.	Students use systematic error analysis to identify potential problems.
		Discussion board.	
Situational awareness	Case presented by video: A young patient dies during induction of a routine anaesthesia due to can’t ventilate, can’t intubate situation.	Interactive video with integrated quiz.	Students apply situational awareness model to identify causes which led to the accident.
		Discussion board.

**Table 2:** Subscales of the German Attitudes to Patient Safety Questionnaire (GAPSQ). Paired results for pre- and post-test data

Scale	Item	*n*	Pre-Test			Post-Test			*p*
			Mean	SD	α	Mean	SD	α	
Patient safety training received		156	4.52	1.61	.81	4.80	1.08	.84	.005
	My training is preparing me to understand the causes of medical errors.	178	4.86	1.31		4.85	1.26		.962
	I have a good understanding of patient safety issues as a result of my undergraduate medical training.	191	4.01	1.40		4.62	1.28		.000
	My training is preparing me to prevent medical errors.	181	4.78	1.28		4.78	1.26		.957
Error reporting confidence		179	4.57	1.46	.83	4.45	1.35	.83	.148
	I would feel comfortable reporting any errors I had made, no matter how serious the outcome had been for the patient.	190	4.88	1.57		4.57	1.47		.001
	I would feel comfortable reporting any errors other people had made, no matter how serious the outcome had been for the patient.	185	4.23	1.50		4.31	1.43		.350
Working hours as error cause (fatigue)		212	6.23	.77	.82	6.41	.82	.93	.001
	Shorter shifts for doctors will reduce medical errors.	219	6.21	.86		6.37	.92		.006
	By not taking regular breaks during shifts, doctors are at an increased risk of making errors.	220	6.35	.90		6.45	.83		.112
	The number of hours doctors work increases the likelihood of making medical errors.	219	6.10	.99		6.38	.87		.000
Error inevitability		223	6.13	.88	.37	6.29	.76	.49	.008
	Even the most experienced and competent doctors make errors.	226	6.51	.76		6.62	.61		.029
	Human error is inevitable.	223	5.75	1.04		5.97	1.19		.038
Patient involvement in reducing error		205	5.16	1.16	.73	5.93	.95	.77	.000
	Patients have an important role in preventing medical errors.	213	4.85	1.35		5.78	1.04		.000
	Encouraging patients to be more involved in their care can help to reduce the risk of medical errors occurring.	215	5.44	1.24		6.09	1.02		.000
Importance of patient safety in the curriculum		198	5.76	.95	.84	5.70	1.11	.80	.341
	Teaching students about patient safety should be an important priority in medical students training.	219	5.67	1.02		5.71	1.18		.582
	Learning about patient safety issues before I qualify will enable me to become a more effective doctor.	201	5.81	.108		5.69	1.25		.126

The post-test contained the same scales for GAPSQ and STS, as well as four knowledge questions comparable to those on the pre-test. To analyse usability and student satisfaction, the post-test contained the system usability scale [44], which is widely tested and accepted for reliability and validity [45]. Overall student satisfaction was measured by asking students to rate each module of the course on a 5-point global rating scale (1 = Not satisfied at all; 5 = very satisfied). Prior to data analysis by RG, the dataset was de-identified by a research assistant, and missing data analysis was performed. No item had to be eliminated from the analysis. For statistical analysis (SPSS, Vers. 23, IBM), we used paired *t*-tests, one-way ANOVA and bivariate correlations. The study is registered with the German Clinical Trials Register, No. DRKS00009762, and the protocol was approved by the Ethics Committee of Freiburg University (reg. no. 59/16). Participants were informed on the aim of the questionnaire and that data would be analysed anonymously.

## Results

### Sample

Three hundred twenty-one students were enrolled in the study; 252 (79 %) participated in the post-test, and complete pre- and post-test data were available for 224 (70 %). The majority (63 %) of the participants were female, and 73 % were between 20 and 24 years old. Most (71 %) of the participants did not have prior experience in the healthcare system; others had experience as nurses (9 %), paramedics (11 %) or other professions including physiotherapy, midwifery and more (9 %).

### Knowledge, values and attitudes

The mean initial knowledge level on patient safety was 36.27 % (SD 23.88). After completion of the e-learning program, the knowledge level increased to 76.45 % (SD 22.97; paired *t*-test, *p* < 0.001; Cohen’s d = 1.72). Similar changes occurred in students’ attitudes towards patient safety: The largest difference related to the benefits of patient involvement: After the intervention, students were more positive about the value of patient involvement (5.16 [SD 1.16] vs. 5.93 [SD .95] vs., *p* < 0.001, Cohen’s d = .73). After the intervention, students acknowledged more readily the effect of fatigue on patient safety (6.23 [SD .77] vs. 6.42 [SD .83] vs., *p* < 0.01, Cohen’s d = .24). Although students were aware before the e-learning course that human error is inevitable (5.75 [SD 1.40]), their ratings increased significantly after the course (5.97 [SD 1.19]; *p* < 0.05, Cohen’s d = .17). Similarly, students strongly agreed that even the most experienced doctors make errors. But that item also was rated significantly higher after the course (6.62 [SD .61] vs. 6.51 [SD .76]; *p* < 0.05; Cohen’s d = .16). Before the online course, students expressed only moderate agreement that they receive sufficient patient safety training in their curriculum (4.52, SD 1.16); after the course, they felt slightly better prepared (4.81 [SD 1.08]; *p* < 0.01; Cohen’s d = .26). The remaining two subscales of the GAPSQ did not show significant pre-post differences. Table 2 summarises all pre- and post-test data for the GAPSQ.

To identify potential differences between those students who had prior healthcare experience and those who did not, we conducted a one-way ANOVA for pre- and post-test data on the GAPSQ variables. In the pre-test data, significant differences emerged on the subscale *fatigue* (F(4, 267) = 2.43; *p* < 0.05). Post hoc analysis using the least significant difference (LSD) method revealed that nurses rated effects of fatigue significantly (*p* < 0.01) higher than did students without medical background. Significant differences were also observed for the statement “Human error is inevitable” in the pre-test data (F(4,283) = 3.07; *p* < 0.05). Paramedics rated this item significantly lower than did other subgroups (*p* < 0.05 for LSD analysis). These pre-course differences levelled out after the course: No significant difference between student groups for any subscale of the GAPSQ was found in the post-test data.

### Systems thinking

Compared with specific knowledge and GAPSQ data, complete datasets for the systems thinking scale were relatively small (*n* = 114). Pre-test scores averaged 58.72 (SD 7.85) on a scale from 0 to 80. After the e-learning course, systems thinking increased significantly, to 61.27 (SD 8.50, *p* < 0.001, Cohen’s d = .31). When analysing correlations between systems thinking and outcome parameters, significant relationships could be identified for the objective parameter of specific post-course knowledge as well as the subjective measure of students’ satisfaction with the e-learning modules. All correlations showed moderate [46] relationships (*r* = .23 to *r* = .36) and were significant at the level of *p* < 0.01 (Table 3).

**Table 3:** Correlation coefficients testing association between systems thinking, specific knowledge and satisfaction with e-learning elements

Measure	Specific knowledge (post)	Satisfaction teamwork module	Satisfaction error management module	Satisfaction podcast situation awareness
Systems thinking score (post)	,264^**^	,361^**^	,309^**^	,230^**^
Specific knowledge (post)		,169^*^	,126	,187^**^
Satisfaction teamwork module			,639^**^	,398^**^
Satisfaction error management module				,326^**^

**p < .01

### Usability and student satisfaction

Students perceive ELPAS as easy to use, this is reflected in a usability score of 73.4 (SD = 16.23). This is equal to an adjective rating of “good usability” according to Bangor et al. [47] Students were satisfied with all modules of the e-learning programme: Satisfaction (1=“not satisfied at all”; 5─“very satisfied”) with the modules on teamwork (4.01, SD .82) and error management (4.05, SD .79) was slightly better than with the situational awareness module (3.74, SD .92). Students did appreciate videos and graphics which facilitate understanding of complex relations. Self-assessment quizzes were considered as very helpful. Some students had difficulties using the etherpad technology for small-group collaboration, however all groups presented well-structured solutions to the scenarios.

## Discussion

After completing an e-learning course on different aspects of patient safety, students’ awareness and knowledge regarding these issues increased. Use of e-learning in medical education is growing and shows promising results, especially for complex topics (such as patient safety), where interactive elements can facilitate learning [47]. Despite these merits, e-learning is hardly used for issues of patient safety yet. In a systematic review, Gordan and colleagues [17] reported on 22 studies measuring the effects of learning interventions on non-technical skills and attitudes towards patient safety. None of these studies used an e-learning intervention. Only recently did McCarthy and colleagues [29] report effects of an online learning program on patient safety; their results were comparable to ours. Our findings shed more light on the potential merits of e-learning programs in patient safety. Not unexpectedly, and still promising, is the large effect of the program for acquisition of specific knowledge on patient safety. Such specific knowledge includes definitions and rules, which are - according to the five-stage model of skills acquisition [30] – the foundation for skills acquisition. Although knowledge alone will hardly improve patient safety, it is considered an important basis for such improvement [48]. Our results suggest that through knowledge acquisition, attitudes change as well: Students perceived patients’ contributions to patient safety as much more important after they completed the online course, although the concept of patient involvement [49] was not even a specific topic of the course. The relatively large effect on the subscale of patient involvement might be a consequence of the systems thinking approach fostered by the online course [26]. Furthermore, since the module *error management* focused on a multi-perspective analysis of errors, the patient’s perspective was also integrated, which in turn may have focused students’ attention towards patient empowerment. Although we could not demonstrate relevant direct relationships between systems thinking and the attitudes on patient safety in our study, we can prove that there is a moderate association to specific knowledge: These results support the hypothesis that through improved systems thinking, a better understanding of patient safety theory can be achieved [20, 31, 36]. Although some relevant attitudes towards patient safety could be altered through the e-learning course, other dimensions, such as error disclosure and reporting, remained unchanged. Error disclosure per se is a difficult topic for physicians, as both ethical [50] and medicolegal [51] consequences are associated with it. Although students involved in this study are not yet responsible for any part of patient care, their willingness to disclose errors was limited initially and did not change after the e-learning course. Other studies show that willingness to disclose errors often decreases with experience [24, 41]. Despite error reporting’s being addressed in the *error management* module of the course, the module focuses more on systems-level incident analysis than on error disclosure. More emphasis, especially on the legal aspects of error disclosure, may be necessary to alter students’ confidence to report errors. White and colleagues have expressed a similar conclusion in regard to error-disclosure training [27].

Because students in this study have limited experience in the healthcare system, we expected to see differences in both knowledge and attitudes when comparing novice students and those who have a medical background. Whereas other studies using the GAPSQ could not find any significant differences [41], in our study, nurses were more aware of the effects of fatigue than were students without prior medical experience. The negative effects of sleep deprivation on patient safety are well documented [52], and it is likely that nurses had accumulated individual experiences during their work in the hospital setting. A reverse effect was present in the subgroup of paramedics: Their rating of the statement “Human error is inevitable” was significantly lower than the ratings of other subgroups. This large difference (4.87 for paramedics vs. 5.78 for students without prior medical training) may be explained by the focused experience of pre-hospital care providers: Processes in this field are highly standardized and based on written guidelines and procedures [53]. Paramedics, who are accustomed to working with such protocols, may experience them as a helpful way to reduce or overcome human error [54].

## Limitations

This study measures self-reported attitudes towards patient safety in students who have limited expertise in the medical field. The longitudinal within-subjects design with pre- and post-course evaluation only detects immediate, short-term effects. Repeated measures may be needed to identify retention of learning and/or stabilisation of attitudes. The generalizability of the results is limited because we only included medical students from Freiburg University and the participation at the online course was mandatory. Because of the voluntary nature of students’ participation, selection bias cannot be ruled out because more motivated students are presumably more likely to participate. Furthermore, the study’s design does not allow for a causal interpretation of the relationships found. Longitudinal or intervention studies should be conducted to examine the causality of the proposed relationships.

## Conclusions

To our knowledge, this study was the first successful attempt in Germany to integrate a patient safety course early in the clinical phase of the medical curriculum. Our results suggest that e-learning technology and methodology can be used for knowledge acquisition on theoretical aspects of patient safety. In this way, face-to-face interventions that are more resource-intensive can be better targeted for action-based learning methodologies. As the e-learning course enhanced the awareness of patient safety, affective outcomes were also achieved. Attitudes towards patient safety improved on several dimensions. Furthermore, we were able to demonstrate that a specifically designed e-learning program can foster the development of conceptual frameworks such as systems thinking, which facilitates the understanding of complex socio-technical systems within healthcare organisations. On the basis of our results, patient safety education will be reinforced in our curriculum. Complementary to hands-on training in later stages of the curriculum, brief e-learning modules will be integrated longitudinally in the curriculum to serve as learning boosters and to increase retention of learned material.

## Abbreviations

ELPAS, e-learning patient safety; GAPSQ, german attitudes to patient safety questionnaire; PS, patient safety; STS, Systems Thinking Scale.
